# The Pattern of Influenza Virus Attachment Varies among Wild Bird Species

**DOI:** 10.1371/journal.pone.0024155

**Published:** 2011-09-01

**Authors:** Elsa Jourdain, Debby van Riel, Vincent J. Munster, Thijs Kuiken, Jonas Waldenström, Björn Olsen, Patrik Ellström

**Affiliations:** 1 INRA, UR 346, Saint Genès Champanelle, France; 2 Department of Virology, Erasmus Medical Center, Rotterdam, The Netherlands; 3 Laboratory of Virology, Rocky Mountain Laboratories, Division of Intramural Research, National Institute of Allergy and Infectious Diseases, National Institutes of Health, Hamilton, Montana, United States of America; 4 Section for Zoonotic Ecology and Epidemiology, School of Natural Sciences, Linnaeus University, Kalmar, Sweden; 5 Section of Infectious Diseases, Department of Medical Sciences, Uppsala University, Uppsala, Sweden; Johns Hopkins University - Bloomberg School of Public Health, United States of America

## Abstract

The ability to attach to host cells is one of the main determinants of the host range of influenza A viruses. By using virus histochemistry, we investigate the pattern of virus attachment of both a human and an avian influenza virus in colon and trachea sections from 12 wild bird species. We show that significant variations exist, even between closely related avian species, which suggests that the ability of wild birds to serve as hosts for influenza viruses strongly varies among species. These results will prove valuable to assess the possibilities of interspecies transmission of influenza viruses in natural environments and better understand the ecology of influenza.

## Introduction

Wild waterbirds are considered to be the fundamental reservoir of influenza A viruses [1]. However, the role played by different waterbird species in the ecology of influenza viruses remains unclear, and important parameters such as the frequency of interspecies transmission are unknown [1]. The host range of influenza viruses is determined by, among other factors, the ability of the virus to attach to sialic acid (SA) residues on the surface of host cells [2]. Because avian viruses preferentially attach to α-2,3-linked SAs whereas human viruses attach to α-2,6-linked SAs, the distribution of α-2,3- and α-2,6- linked SAs on surface epithelia is usually studied by using plant lectins, such as the *Maackia amurensis* agglutinin (MAA) and the *Sambucus nigra* agglutinin (SNA), which respectively recognize α-2,3- and α-2,6-linked SAs [2], [3], [4]. Lectin studies conducted on bird tissues suggest that SA expression varies depending on species and tissues [5], [6], [7], [8], [9].

However, lectin histochemistry has inherent limitations in predicting host-virus interactions. Indeed, MAA and SNA lectins do not detect differences involving the inner chain of SAs [10], [11] and MAA binding specificity may differ depending on isotypes, with unspecific binding to unsialylated glycoconjugates as well as binding to α2,3-sialylated structures of limited/unknown role in the attachment of influenza viruses [3], [4], [12], [13]. Therefore, the use of labeled influenza virus particles to study the pattern of virus attachment (PVA) in target tissues is a better tool to determine whether a species is receptive to influenza virus infection, and to assess the cell and tissue tropism of viruses [6], [14], [15].

A previous study, comparing human and avian influenza virus PVAs on the upper and lower respiratory tract of humans and several other mammal species [15], identified differences in cell and tissue tropism between human and avian influenza viruses and showed that the PVA in the human respiratory tract corresponds to the main presentation of the disease [15]. The objective of the present study was to determine the PVA of avian and human influenza A viruses in the colon and trachea of wild bird species. Our hypothesis was that differences in PVA could help classify avian species with regard to their roles in the ecology of low pathogenic avian influenza viruses (LPAIVs).

As representatives of influenza viruses with an avian-like and a human-like receptor specificity, we respectively used a LPAIV (H6N1 A/Mallard/Sweden/81/02), which does not attach to human trachea [15], and a seasonal human influenza virus (H3N2 A/Netherlands/213/03), which attaches abundantly to human and pig trachea [15]. We chose the colon and trachea as target tissues because LPAIVs are known to replicate in these tissues in mallards and related domestic ducks [16], [17]. We selected six waterbird species (Table 1) that share similar habitats and from which LPAIVs have been isolated [1]. For comparison, we examined tissues from domestic chickens and six terrestrial species, including three species (rock pigeon, hooded crow, house sparrow) suspected of acting as bridge species for the transmission of avian influenza viruses between wild waterfowl and domestic poultry [18], and three insectivorous species (European robin, goldcrest, blue tit) unlikely exposed to influenza viruses.

**Table 1:** Pattern of virus attachment of an avian influenza virus and a human seasonal influenza virus in the trachea and colon of 12 wild bird species and domestic chicken.

Habitat type	Taxonomic order	Species	Latin name	Prevalence^a^ (%)[95% binomial confidence interval]	Avian virus		Human virus	
					Colon	Trachea	Colon	Trachea
Aquatic	Anseriformes	Mallard	*Anas plathyrhynchos*	12.9 [12.4–13.4]	+++	+++	−	−
		Eurasian wigeon	*Anas penelope*	0.8 [0.4–1.5]	±	+++	+^c^	−
		Greylag goose	*Anser anser*	1.1 [0.6–2.0]	++	+++	++^c^	−
		Tufted duck	*Aythya fuligula*	1.1 [0.2–3.3]	−	+++	−	+
	Charadriiformes	Herring gull	*Larus argentatus*	1.4 [0.7–2.5]	+	+	−	++
	Pelecaniformes	Great cormorant	*Phalacrocorax carbo*	0.4 [0.2–0.6]	±^b^	+++	±	−
Terrestrial	Galliformes	Domestic chicken	*Gallus gallus*		++	+++	+^c^	±
	Columbiformes	Rock pigeon	*Columba livia domesticus*		−	−	−	+++
	Passeriformes	Hooded crow	*Corvus corone cornix*		+	+++	−	−
		House sparrow	*Passer domesticus*		+	−d	±^e^	−
		European robin	*Erithacus rubecula*		++	+++	±	+++
		Goldcrest	*Regulus regulus*		+	±	−	−d
		Blue tit	*Cyanystes caeruleus*		±	+	±^e^	−d

Scoring indicates attachment to ciliated cells for the trachea and intestinal epithelial cells from the crypts or villae for the colon.

−: no significant attachment.

±: attachment to rare or few cells.

+: attachment to a moderate number of cells.

++: attachment to many cells.

+++: attachment to a large majority of cells.

a : prevalence data are extracted from Olsen et al. 2006 [1] and are calculated considering altogether all low pathogenic avian influenza subtypes.

b : attachment to intestinal epithelial cells in 1 of the 3 cormorants.

c : attachment to intestinal epithelial cells in the crypts.

d : attachment to tracheal goblet cells.

e : attachment to colon goblet cells.

## Results and Discussion

### Attachment of the avian virus

**Figure 1:**
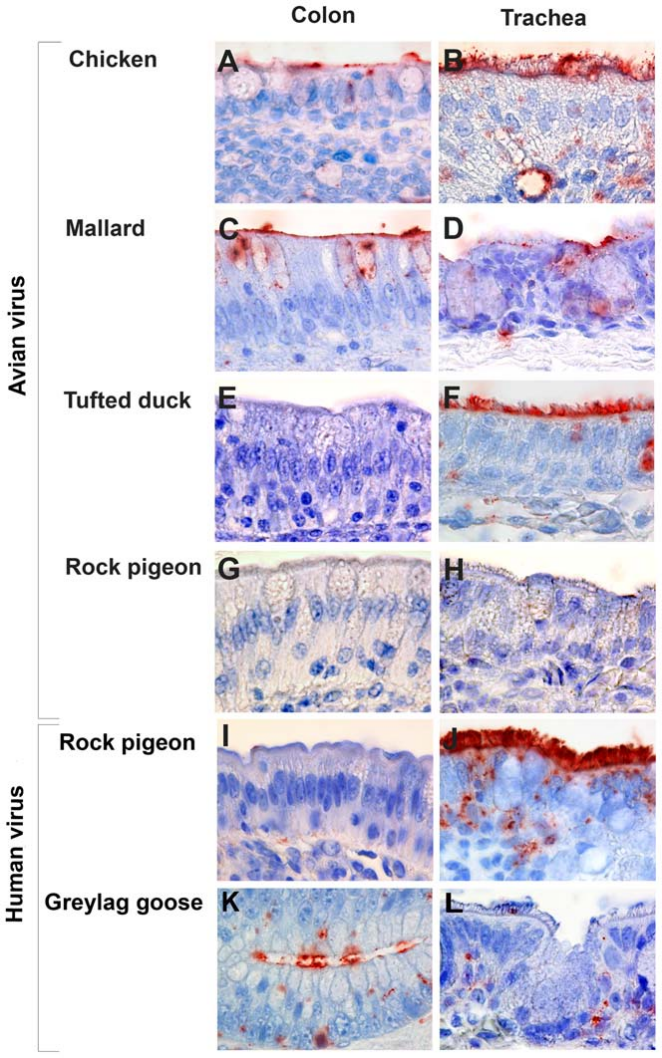
Colon and trachea sections from selected wild bird species showing that the pattern of attachment of avian and human influenza viruses varies between bird species. (a) chicken colon with avian virus; (b) chicken trachea with avian virus; (c) mallard colon with avian virus; (d) mallard trachea with avian virus; (e) tufted duck colon with avian virus; (f) tufted duck trachea with avian virus; (g) rock pigeon colon with avian virus; (h) rock pigeon trachea with avian virus; (i) rock pigeon colon with human virus; (j) rock pigeon trachea with human virus; (k) greylag goose colon crypt with human virus; (l) greylag goose trachea with human virus.

The overall PVA was consistent among individuals of the same bird species. As expected, the avian virus attached to tissues from wild birds more frequently than the human virus (Table 1). The avian virus attached abundantly to both the trachea and colon of mallards and chickens (Figure 1), which corroborates earlier observations of α2,3-linked SAs and avian virus attachment in tracheal and intestinal cells of chickens and mallards [6], [7], [19] and fits with the observation that LPAIV infection results in virus replication in both the respiratory and digestive tracts of these species [16], [20].

The main purpose of this study was to investigate wild bird species other than mallards. Because all waterbird species investigated were reported to carry influenza viruses [1], we expected the avian influenza virus to attach to their colon surface epithelium. Attachment to many cells was observed for geese and European robin, and attachment to a moderate number of cells was observed for gulls and several terrestrial species. However, attachment to only a few goblet cells was detected in wigeons and no attachment was observed in the colon of tufted ducks (Figure 1). This result suggests that the susceptibility of tufted ducks to intestinal infection with this avian virus is relatively low. Given that epidemiological surveys have shown that tufted ducks occasionally shed LPAIVs [1], experimental infections combined with PVA studies should be conducted to assess whether other avian viruses can replicate in their digestive tract.

The detection of significant virus attachment to tracheal ciliated cells of all bird species, except pigeons and sparrows, suggests that the upper respiratory tract may be another major entry route for influenza viruses in wild bird species. These results are consistent with surveillance [21], [22], [23] and experimental [24], [25] studies showing that LPAIVs are frequently detected in oropharyngeal swabs from wild waterfowl as well as lectin studies suggesting that α-2,3-linked SAs are expressed on domestic bird trachea and bronchus [6], [9].

The fact that no avian virus attachment was detected on the colon and trachea of domestic pigeons (Figure 1) suggests that pigeons have low susceptibility to infection by this avian influenza virus. This result fits with observations that H5N1 highly pathogenic avian influenza virus (HPAIV) replication in pigeons only is observed with high inoculation doses [26] and that α-2,3-linked SAs are expressed poorly in pigeons [27].

### Attachment of the human virus

The overall lack of attachment of the human influenza A virus is consistent with a lack of evidence of human influenza virus infection in birds. Exceptions were observed for intestinal epithelial cells from the colon crypts of chickens, wigeons, and geese (Figure 1), and for tracheal ciliated cells of tufted ducks, gulls, robins, and pigeons (Figure 1). Experimental infection is needed to determine whether these avian species are susceptible to human influenza virus infection. If infection by both a human and an avian influenza virus proved possible in these species, then they could be considered, along with domestic pig (*Sus scrofa*) [28] and Japanese quail (*Coturnix coturnix*) [7], as potential mixing vessels for the generation of human-avian reassortants. However, one would expect that, if such infections with human viruses were possible, they already would have been detected, at least for species in very close contact with humans such as chickens, pigeons and geese. A recent experimental inoculation of chickens with human H1N1 influenza A viruses showed very limited infection [29].

### Conclusions

Our results suggest that cell and tissue tropisms are important in determining the host species range of influenza viruses in birds. We found that the PVA of LPAIVs differs between wild bird species, even between species from the same taxonomic family (i.e. with shared phylogenetic origins), and between species with similar feeding behavior (i.e. with a similar risk of ingesting virus particles while feeding). The screening for virus attachment was more discriminating than lectin histochemistry, which has been used to detect the α-2,6-linked SA receptor pattern on wild bird tracheas [5], and proved useful in identifying species with unexpected attachment patterns.

Because the ability of a virus to replicate in cells does not depend solely on attachment, experimental inoculation studies are needed to determine whether viruses can replicate. However, determining differences in PVA between different viruses and bird species is a valuable preliminary screening method. Because we cannot exclude the possibility that other avian and human influenza viruses show a different PVA than those used in this study, we encourage further PVA studies using various influenza A virus isolates and tissues from a broader range of bird species including gulls, which are known to host specific virus subtypes [30], [31]. Knowledge about the PVA of HPAI H5N1 virus in wild bird tissues would be helpful in identifying wild bird species that could potentially spread this zoonotic virus. In the human respiratory tract, the PVA of two LPAIVs (H5N9 and H6N1) and HPAI H5N1 virus did not differ [15]. Whether similar results would be observed with avian tissues remains to be determined.

## Materials and Methods

Tissue sampling procedures were approved by the Swedish Environmental Protection Agency (permits number 412-6267-08NV and 412-5977-08NV) and the Swedish Board of Agriculture (permits number 74-08 and 43-09).

For each species, three individual birds were euthanized humanely and both trachea and colon were sampled within three minutes after death. Tissues were fixed in formalin for 48 h and paraffin-embedded. For each bird and tissue, three transverse sections were incubated with the human or avian virus labeled with fluorescein isothiocyanate (FITC; Sigma-Aldrich, Stockholm, Sweden) [15]. For each tissue tested, in each run an omission control was included to check for unspecific staining and sections from pig trachea (displaying α2,6-linked SAs) and mallard colon (displaying α2,3-linked SAs) were included as positive control tissues, respectively for the human and the avian virus.

We used H6N1 A/Mallard/Sweden/81/02 and H3N2 A/Netherlands/213/03 as representatives of LPAIV and seasonal human influenza virus, respectively. The specificity of the avian virus for α2,3-linked SAs was confirmed by the hemagglutination of horse erythrocytes expressing α2,3-linked SAs only. The specificity of the human virus for α2,6-linked SAs was confirmed by the hemagglutination of modified turkey erythrocytes expressing α2,6-linked SAs only, as described by Chutinimitkul et al. [32].

Virus attachment on tissue sections was detected by virus histochemistry as described previously [14], [15]. Briefly, formalin-fixed paraffin-embedded tissues were deparaffinized with xylene and rehydrated with graded alcohol. FITC-labeled influenza viruses (50 hemagglutination units) were incubated with tissues overnight at 4°C. The FITC label was detected with a peroxidase-labeled rabbit anti-FITC antibody (ab 19492-500, Abcam, Cambridge, UK), and the signal was amplified with a tyramide signal amplification system (Perkin-Elmer, Upplands Väsby, Sweden) according to the manufacturer's instructions. Peroxidase was revealed with 3-amino-9-ethyl-carbazole (Sigma-Aldrich), and tissues were counterstained with hematoxylin and embedded in glycerol-gelatin (Merck, Darmstadt, Germany). Attachment of influenza virus to tissues was visible as granular to diffuse red staining on the apical surface of epithelial cells. We considered that the most important cells for virus attachment were ciliated cells in the trachea and intestinal epithelial cells from crypts and villi in the colon [16], [17]. The proportion of cells to which the virus attached was scored as follows: - no significant attachment, ± attachment to rare or few cells, + attachment to a moderate number of cells, ++ attachment to many cells, +++ attachment to a large majority of cells.
